# Tricin inhibits the migration of human retinal pigment epithelium cells by suppressing the RUNX2-CYP1A1 axis and STAT3 pathway

**DOI:** 10.7150/ijms.122814

**Published:** 2025-10-20

**Authors:** Wei-Yang Lu, I-Chia Liang, Yi-Hsien Hsieh, Kai Wang, Chia-Yi Lee, Nuo-Yi Yu, Shun-Fa Yang, Hsiang-Wen Chien

**Affiliations:** 1 Department of Ophthalmology, Changhua Christian Hospital, Changhua, Taiwan.; 2 Institute of Medicine, Chung Shan Medical University, Taichung, Taiwan.; 3 Department of Optometry, Chung Shan Medical University, Taichung, Taiwan.; 4 Department of Ophthalmology, Tri-Service General Hospital, National Defense Medical Center, Taipei, Taiwan.; 5 Department of Ophthalmology, Cathay General Hospital, Taipei, Taiwan.; 6 Department of Medical Research, Chung Shan Medical University Hospital, Taichung, Taiwan.; 7 Departments of Ophthalmology, Sijhih Cathay General Hospital, New Taipei City, Taiwan.; 8 School of Medicine, College of Medicine, Fu Jen Catholic University, New Taipei, Taiwan.; 9 School of Medicine, National Tsing Hua University, Hsinchu, Taiwan.

**Keywords:** tricin, retinal pigment epithelium, proliferative vitreoretinopathy, CYP1A1, RUNX2, STAT3

## Abstract

Proliferative vitreoretinopathy (PVR) is a retinal disorder characterized by abnormal growth and migration of retinal pigment epithelium (RPE) cells, leading to impaired visual acuity. Tricin is a naturally occurring flavone known to inhibit the migration of various cancer cell types. Therefore, the aim of this study was to investigate the potential inhibitory effects of tricin on the migration of ARPE-19 cells. In this study, tricin treatment significantly reduced the migratory and invasive abilities of ARPE-19 cells in the Boyden chamber assays. RNA sequencing identified cytochrome P450 1A1 (CYP1A1) as the most significantly downregulated gene following tricin treatment. Real-time PCR confirmed a reduction in CYP1A1 mRNA levels, while Western blot analysis demonstrated a concentration-dependent decrease in CYP1A1 protein expression. Moreover, siRNA-mediated knockdown of CYP1A1 resulted in decreased mRNA expression levels, accompanied by reduced cell migration. Tricin treatment also attenuated RUNX2 transcription factor levels and phosphorylation of STAT3. Co-treatment with tricin and colivelin (a STAT3 activator) led to increased CYP1A1 expression and enhanced cell migration, suggesting a regulatory role of the STAT3 pathway in tricin-mediated effects. In conclusion, tricin inhibits the migration of ARPE-19 cells by downregulating CYP1A1 and RUNX2 expression through suppression of the STAT3 signaling pathway. These findings suggest that tricin holds potential as a therapeutic candidate for preventing or limiting the progression of PVR.

## Introduction

Proliferative vitreoretinopathy (PVR) is an ocular condition characterized by the formation and contraction of fibrotic membranes throughout the intraretinal space, vitreous cavity, and retinal surface 1, 2. PVR is a major cause of surgical failure in retinal detachment repair, contributing to poor anatomical outcomes in approximately 10% of cases 3. The primary intervention for PVR is trans pars plana vitrectomy; however, postoperative outcomes often remain unsatisfactory 4. Several predisposing factors have been identified, including uveitis, retinal tears, vitreous hemorrhage, and prior vitrectomy procedures 5.

The pathogenesis of PVR is primarily driven by the migration and proliferation of retinal pigment epithelium (RPE) cells, which invade the vitreous cavity 1. Under normal physiological conditions, RPE cells reside beneath the neurosensory retina and the Bruch's membrane and exhibit minimal turnover 3. However, pathological conditions lead to RPE proliferation and epithelial-mesenchymal transition (EMT), which are stimulated by a variety of cytokines, including platelet-derived growth factor (PDGF) and insulin-like growth factor (IGF) 6, 7. Furthermore, intracellular signaling pathways such as extracellular signal-regulated kinases 1 and 2 (ERK1/2) and c-Jun N-terminal kinases 1 and 2 (JNK1/2) have been shown to mediate RPE transformation and migration 8. In addition, other compounds such as dihydromyricetin, demethoxycurcumin, and protein kinase Cα inhibitors have been shown to regulate RPE cell behavior 9-11.

Tricin, a naturally occurring flavonoid, has been shown to influence cellular signaling and inhibit cell proliferation 12-14. Tricetin, a structurally related flavonoid, has been reported to suppress glioma cell migration 15, and reduce the proliferation of human hepatic stellate cells 16. Additionally, our previous study reported that tricetin suppresses RPE cell migration and BMP-6 expression through the p38 signaling pathway 17. However, limited studies have investigated the effect of tricin on RPE cell migration. Given the structural similarity between tricin and tricetin 18, the tricin may own certain influence on the RPE cell migration which need verification. Therefore, the aim of the present study was to investigate the effect of tricin on RPE cell migration and to explore the molecular pathways involved in this process.

## Materials and Methods

### Chemicals and reagents

Tricin was purchased from ChromaDex (Longmont, CO, USA). A stock solution of 40 mM tricin was prepared in dimethyl sulfoxide (DMSO; Sigma-Aldrich, St. Louis, MO, USA) and stored at -20 °C. The final concentration of DMSO in all experimental conditions was maintained below 0.5%.

### Cell lines

The RPE cell line used in this study, ARPE-19, is a spontaneously arising human retinal pigment epithelial cell line derived from a 19-year-old male donor. ARPE-19 cells were obtained from the American Type Culture Collection (ATCC, Manassas, VA, USA) and cultured in Dulbecco's Modified Eagle's Medium/Nutrient Mixture F-12 (DMEM/F12; HyClone, Logan, UT, USA), supplemented with 10% fetal bovine serum (FBS), under a humidified atmosphere of 5% CO₂ at 37 °C.

### Cell viability assay

To assess the cytotoxicity of tricin, ARPE-19 cells were seeded in 24-well plates and treated with different concentrations of tricin (0, 10, 20, and 40 μM) for 24 hours at 37 °C. Subsequently, 0.5 mg/mL of MTT reagent (3-(4,5-dimethylthiazol-2-yl)-2,5-diphenyltetrazolium bromide; Sigma-Aldrich) was added to each well and incubated for an additional 4 hours. The resulting formazan crystals were dissolved in isopropanol, and absorbance was measured at 563 nm using a spectrophotometer.

### Boyden chamber assay for cell migration

ARPE-19 cells were pretreated with tricin (0, 10, 20, and 40 μM) for 24 hours and then seeded (1 × 10⁵ cells/well) into the upper chambers of a Boyden chamber (Neuro Probe, Cabin John, MD, USA) containing serum-free medium 19. After incubation at 37 °C for 24 hours, the number of migrated cells was evaluated. Additionally, the same setup was used to assess the invasive capability of ARPE-19 cells following treatment with tricin.

### Real-time polymerase chain reaction (PCR) for genes in ARPE-19

Total RNA was extracted using the Total RNA Mini Kit (Geneaid, New Taipei City, Taiwan), and reverse-transcribed into complementary DNA (cDNA) using the High-Capacity cDNA Reverse Transcription Kit (Applied Biosystems, Thermo Fisher Scientific, Chino, CA, USA). Quantitative real-time PCR was performed using Fast SYBR Green Master Mix (Applied Biosystems, Thermo Fisher Scientific, Chino, CA, USA) and specific primers on the StepOnePlus Real-Time PCR System (Applied Biosystems, Thermo Fisher Scientific, Chino, CA, USA). Cycle threshold (Ct) values were used to quantify gene expression, which was normalized against internal controls.

### Western blot analysis

ARPE-19 cells were treated with tricin (0, 10, 20, and 40 μM) for 24 hours. Total cell lysates were prepared using the Mammalian Protein Extraction Buffer Kit containing phosphatase inhibitors, as previously described 20. Proteins were separated by 10% SDS-PAGE and transferred onto polyvinylidene fluoride (PVDF) membranes. The membranes were incubated with specific primary antibodies, followed by HRP-conjugated secondary antibodies. Protein bands were detected using enhanced chemiluminescence (ECL) with Immobilon Western HRP Substrate (Millipore, Billerica, MA, USA).

### Small interfering RNA (siRNA) analysis for ARPE-19 cell migration

To inhibit CYP1A1 expression, non-targeting siRNA and four CYP1A1-specific siRNAs were used (GenePharma Co., Ltd, Shanghai, China). ARPE-19 cells were seeded in 6-well plates and incubated for 24 hours, followed by transfection with the respective siRNAs using Lipofectamine RNAiMAX reagent (Invitrogen, Carlsbad, CA, USA) for 48 hours. After transfection, cell migration was assessed using the Boyden chamber assay.

### Statistical analysis

All statistical analyses were performed using SAS software (version 9.4; SAS Institute Inc., Cary, NC, USA). An independent t-test was used for comparisons between groups. A p-value < 0.05 was considered statistically significant.

## Results

### Effects of Tricin on the viability, migration, and invasion of ARPE-19 cells

**Figure 1:**
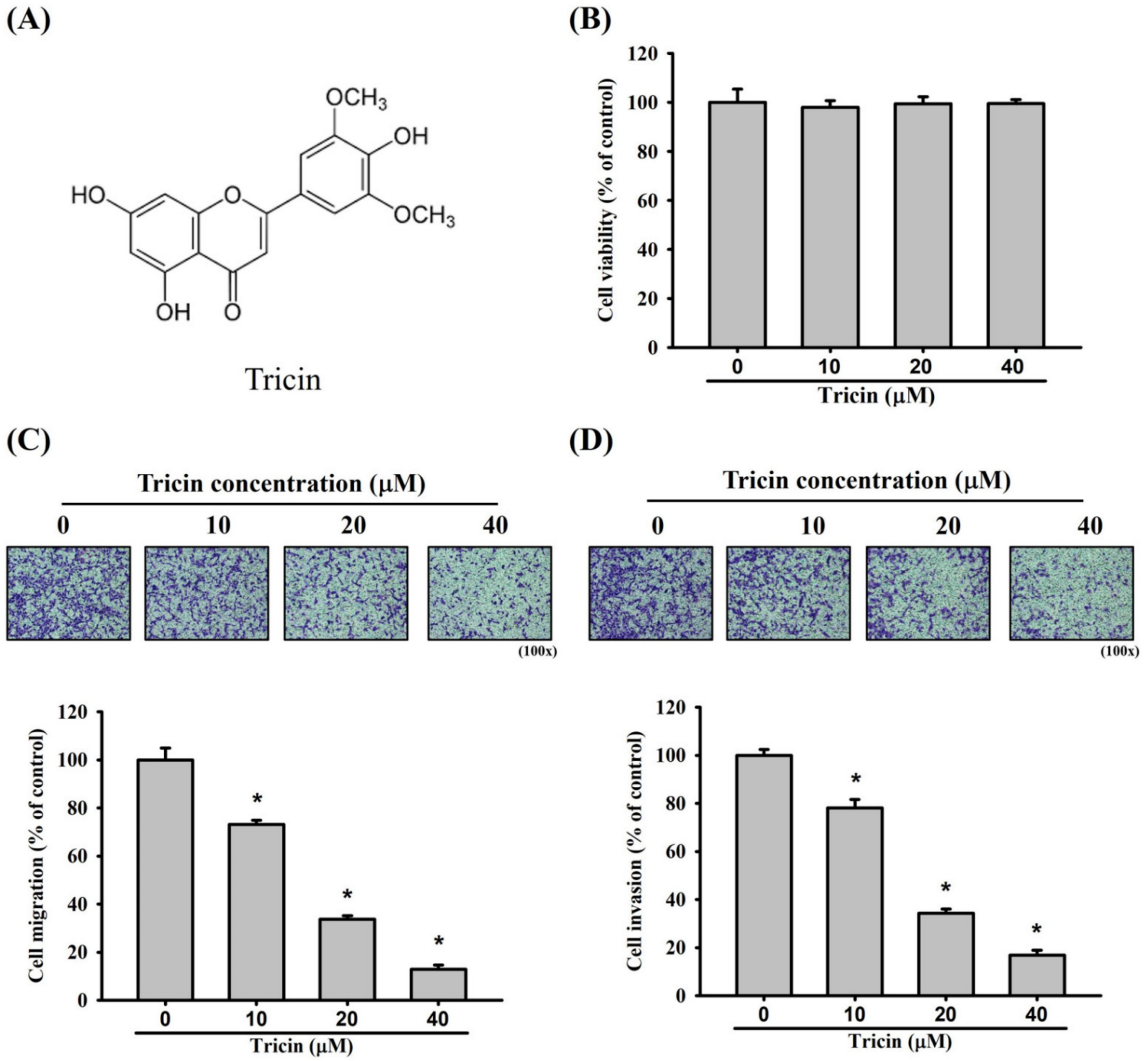
** Tricin and its effect on ARPE-19 cell viability, cell migration and invasion.** (A) Chemical structure of tricin. (B) Cell viability of ARPE-19 cells following treatment with different concentrations of tricin (0, 10, 20, and 40 μM) for 24 hours, as assessed by the MTT assay. (C) Migration of ARPE-19 cells treated with various concentrations of tricin (0, 10, 20, and 40 μM) for 24 hours, evaluated using the Boyden chamber migration assay. (D) Invasion of ARPE-19 cells treated with various concentrations of tricin (0, 10, 20, and 40 μM) for 24 hours, analyzed using the Boyden chamber invasion assay. *Indicates statistically significant difference (p < 0.05).

The chemical structure of tricin is shown in Figure 1A. Treatment with varying concentrations of tricin did not significantly affect the viability of ARPE-19 cells (Figure 1B). Nevertheless, in the Boyden chamber assay, tricin treatment significantly reduced the migratory capacity of ARPE-19 cells compared to the control group at all tested concentrations (Figure 1C). Similarly, tricin-treated ARPE-19 cells exhibited significantly decreased invasive ability in the Boyden chamber invasion assay compared to controls (Figure 1D).

### Gene expression profiling of tricin-treated ARPE-19 cells

**Figure 2:**
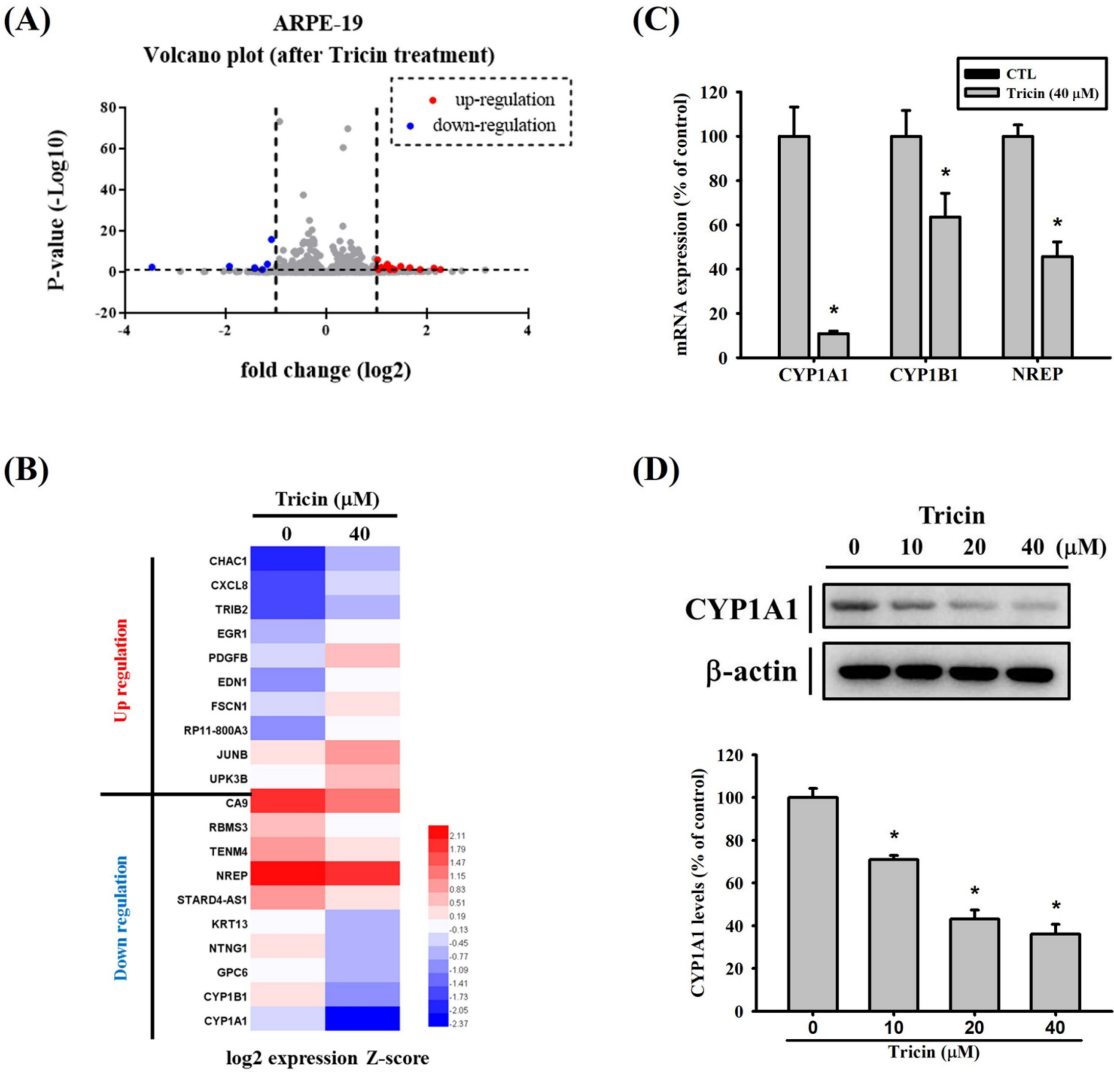
** CYP1A1 gene expression in ARPE-19 cells following tricin treatment.** (A) Volcano plot showing differentially expressed genes in ARPE-19 cells after treatment with 40 μM tricin for 24 hours. (B) Hierarchical clustering heatmap of 20 selected genes based on RNA sequencing of ARPE-19 cells treated with 40 μM tricin for 24 hours. (C) Quantitative real-time PCR analysis of CYP1A1, CYP1B1 and NREP mRNA expression in ARPE-19 cells treated with 40 μM tricin for 24 hours. (D) Western blot analysis of CYP1A1 protein expression in ARPE-19 cells treated with indicated concentrations of tricin for 24 hours. β-actin was used as a loading control. *Indicates statistically significant difference (p < 0.05).

**Figure 3:**
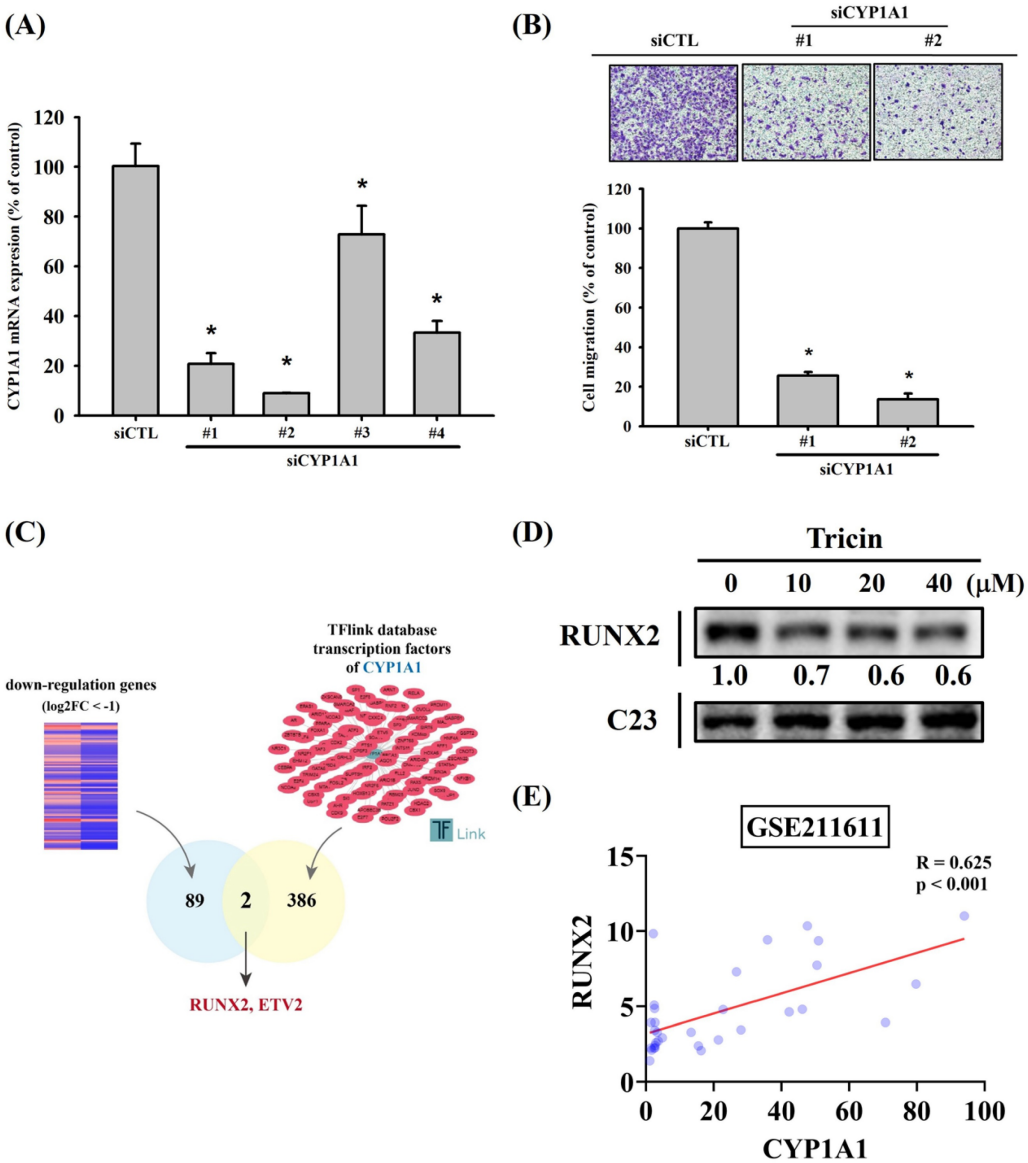
** siRNA-mediated knockdown of CYP1A1 in ARPE-19 cells.** (A) CYP1A1 mRNA expression levels in ARPE-19 cells transfected with different CYP1A1-specific siRNAs compared to the control. (B) Migration ability of ARPE-19 cells after treatment with different CYP1A1 siRNAs, assessed using the Boyden chamber assay. *Indicates statistically significant difference (p < 0.05). (C) The diagrams depict the selection of transcription factor candidates targeting CYP1A1. (D) Western blot analysis of RUNX2 protein expression in ARPE-19 cells treated with the indicated concentrations of tricin for 24 hours. C23 was used as a loading control. (E) Correlation analysis of RUNX2 and CYP1A1 expression in human ARPE-19 cells using the GSE211611 dataset.

To investigate the effects of tricin on gene expression, RNA sequencing was performed. The volcano plot illustrating differentially expressed genes in tricin-treated ARPE-19 cells is shown in Figure 2A, and a heat map of significantly upregulated and downregulated genes is presented in Figure 2B. Among all the genes affected by tricin, CYP1A1 was the most significantly downregulated (log2 fold change: -3.46, Table 1). To validate these findings, real-time PCR and Western blot analyses were conducted. Real-time PCR confirmed a significant decrease in CYP1A1 mRNA expression following tricin treatment (Figure 2C), and Western blot revealed a corresponding dose-dependent reduction in CYP1A1 protein levels (Figure 2D). Furthermore, siRNA-mediated knockdown of CYP1A1 resulted in a significant decrease in CYP1A1 mRNA levels, as well as reduced migratory ability of ARPE-19 cells (Figure 3A-B). To investigate which transcription factors are involved in tricin-mediated inhibition of CYP1A1, bioinformatic analysis was performed using the TFLink database. By integrating the differentially expressed genes (DEGs) identified in tricin-treated ARPE-19 cells, RUNX2 and ETV2 were identified as potential candidates (Figure 3C). In addition, Western blot analysis demonstrated that tricin treatment significantly reduced RUNX2 expression in ARPE-19 cells (Figure 3D). Furthermore, analysis of the GSE211611 dataset from the Gene Expression Omnibus (GEO) revealed a positive correlation between CYP1A1 and RUNX2 expression in human ARPE-19 cells (Figure 3E). Collectively, these findings indicate that the suppression of CYP1A1 and RUNX2 expression may contribute to the inhibitory effect of tricin on ARPE-19 cell migration.

### Tricin modulates STAT3 signaling in ARPE-19 cells

**Figure 4:**
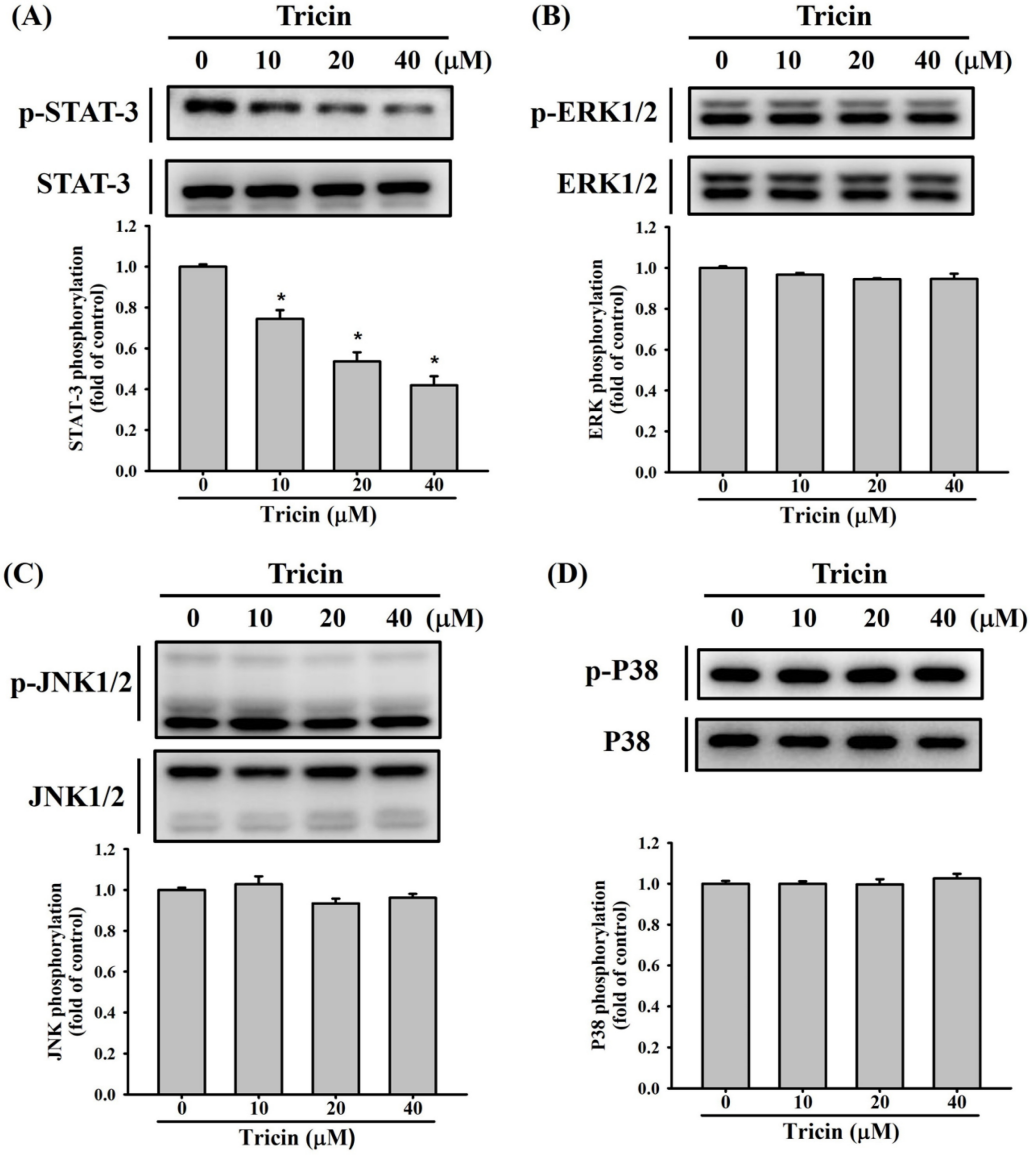
** Tricin modulates expression of STAT3, ERK1/2, JNK1/2 and p38 signaling pathways in ARPE-19 cells.** (A) Western blot analysis of total and phosphorylated STAT3 in ARPE-19 cells treated with different concentrations of tricin (0, 10, 20, and 40 μM) for 24 hours. *Indicates statistically significant difference (p < 0.05). (B) Western blot analysis of total and phosphorylated ERK1/2 under the same treatment conditions. (C) Western blot analysis of total and phosphorylated JNK1/2. (D) Western blot analysis of total and phosphorylated p38 MAPK.

Previous studies have reported that STAT3 and the MAPK signaling pathways, including ERK1/2, JNK1/2, and p38 kinases, constitute a central regulatory cascade involved in RPE cell migration 8, 9, 17, 21. To explore the effect of tricin on STAT3 and the MAPK signaling pathways involved in ARPE-19 cell behavior, Western blot analysis was conducted. Tricin treatment led to a dose-dependent decrease in STAT3 phosphorylation (Figure 4A), while phosphorylation levels of ERK1/2, JNK1/2, and p38 remained unchanged (Figure 4B-D).

**Figure 5:**
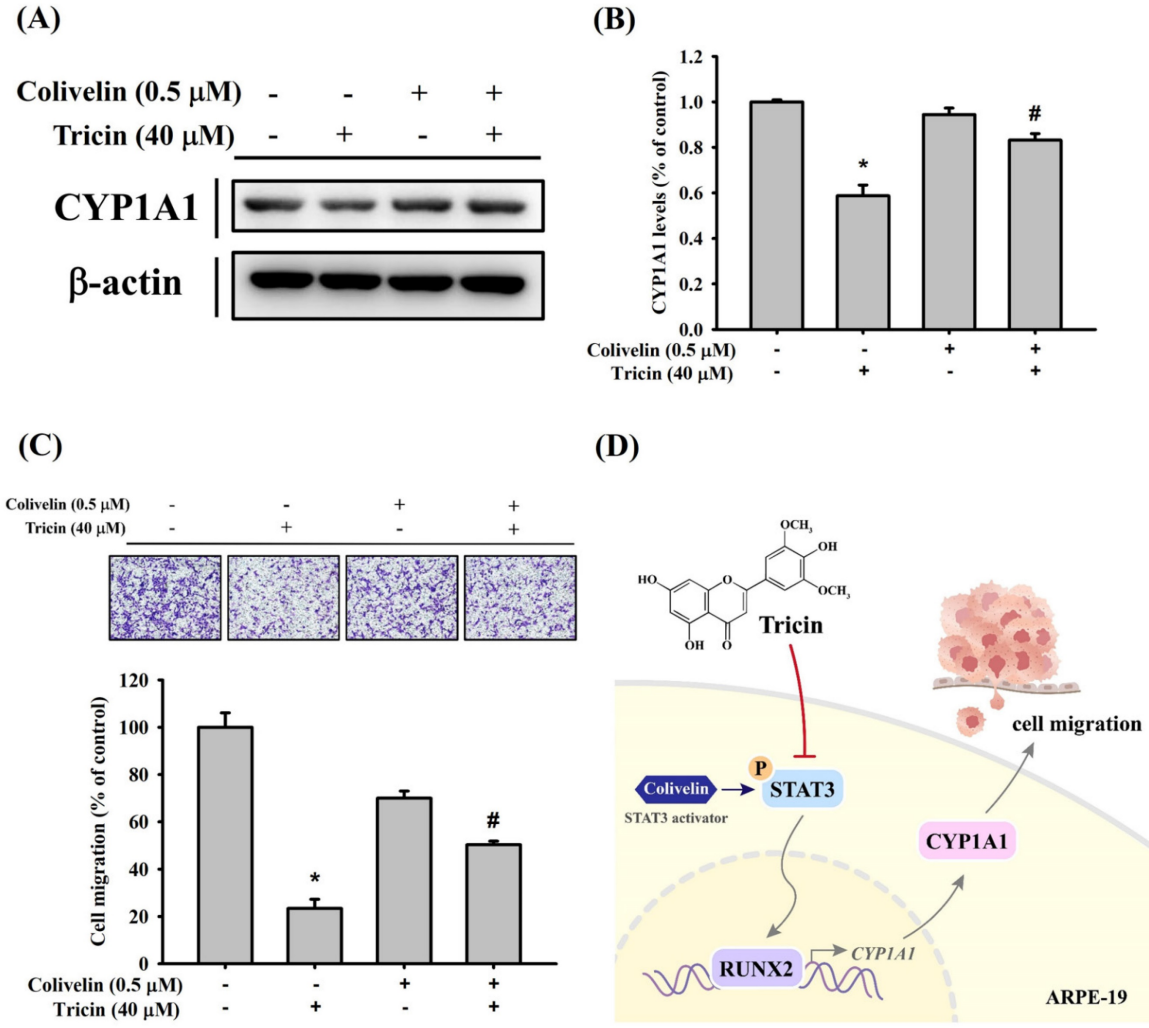
** Effect of STAT3 activator (colivelin) on CYP1A1 expression and cell migration in ARPE-19 cells.** (A) Western blot analysis of CYP1A1 protein expression in ARPE-19 cells treated with or without 40 μM tricin and 0.5 μM colivelin for 24 hours. β-actin was used as a loading control. (B) Densitometric analyses of immunoblots were performed by the ImageJ software. (C) Migration of ARPE-19 cells treated with or without 40 μM tricin and 0.5 μM colivelin for 24 hours, evaluated using the Boyden chamber assay. *p < 0.05, compared with the vehicle group. #p < 0.05, compared with the colivelin treated group. (D) Schematic diagram for proposed signaling pathways in the inhibitory mechanisms of tricin on cell migration in human ARPE-19 cells.

To further validate the relationship between STAT3 signaling and CYP1A1 expression under tricin treatment, cells were co-treated with tricin and colivelin (a STAT3 activator). Western blot results demonstrated that colivelin co-treatment restored CYP1A1 expression compared to tricin treatment alone (Figure 5A-B). Additionally, Boyden chamber analysis showed restored migration in cells treated with both tricin and colivelin compared to tricin alone (Figure 5C), suggesting that STAT3 activity is involved in tricin-mediated suppression of CYP1A1 and cell migration.

## Discussion

In this study, tricin treatment significantly reduced the migration and invasion of ARPE-19 cells. This effect was accompanied by a marked downregulation of CYP1A1 and RUNX2 expression, which was correlated with reduced cell motility. Furthermore, tricin suppressed phosphorylation of STAT3, suggesting that this signaling pathway may mediate the tricin-induced downregulation of CYP1A1.

Flavonoids have been widely reported to modulate cell migration, proliferation, and signaling pathways, playing roles in a variety of pathological processes 18, 22, 23. For instance, tricetin, a structurally related flavonoid, was shown to inhibit the migration of human glioblastoma multiforme cells by downregulating the matrix metalloproteinase-2 (MMP-2) pathway 23, and to suppress the migration of human oral cancer cells through inhibition of the MAPK signaling cascade 18. It has also been reported to reduce the migration of nasopharyngeal carcinoma cells 24. Additionally, tricin and its derivatives exhibit anti-proliferative effects in cancer models, including non-small cell lung cancer 25, osteosarcoma 22, and leukemia 26, primarily by inducing apoptosis or cell cycle arrest.

In the context of RPE cells, numerous factors regulate their proliferation and migration. For example, growth factors such as PDGF and HTRA1 have been implicated in RPE proliferation and PVR pathogenesis 27, 28. Conversely, compounds like dihydromyricetin and PKC-α antagonists have been shown to reduce the migration and invasion of ARPE-19 cells 11. Given the structural and functional similarities between tricin and tricetin, and their known effects on other cell types, 17, 18, we hypothesized that tricin may similarly suppress the migratory capacity of RPE cells. The present study supports this hypothesis by demonstrating a significant and dose-dependent reduction in ARPE-19 cell migration and invasion following tricin treatment.

In this study, gene expression analysis revealed CYP1A1 as the most significantly downregulated gene in tricin-treated ARPE-19 cells. CYP1A1 encodes an enzyme in the cytochrome P450 family involved in the metabolism of drugs, xenobiotics, and endogenous substrates 29. It has also been implicated in the metabolic activation of procarcinogens 30. Elevated CYP1A1 expression has been associated with cancer progression, and its genetic polymorphisms are linked to increased risks of breast and lung cancers 31-33. Moreover, CYP1A1 has been implicated in ocular neovascular diseases such as choroidal neovascularization 34. In this study, CYP1A1 knockdown via siRNA similarly reduced ARPE-19 cell migration, suggesting that CYP1A1 plays a functional role in RPE motility and is a key downstream target of tricin.

Mechanistically, tricin inhibited phosphorylation of STAT3 in a concentration-dependent manner, while having minimal effects on the ERK1/2, JNK1/2, and p38 pathways. Furthermore, co-treatment with colivelin, a STAT3 activator, restored CYP1A1 expression and increased cell migration, supporting the involvement of the STAT3-CYP1A1 axis in tricin's effects. Although little prior work has explored this mechanism, previous studies have shown that STAT3 signaling promotes ARPE-19 cell migration and EMT. For instance, demethoxycurcumin inhibited ARPE-19 cell migration by suppressing STAT3 phosphorylation 9, and silibinin reduced EMT through STAT3 pathway 35. Collectively, these findings suggest that STAT3 is a central regulatory pathway in ARPE-19 cell behavior and a viable target for therapeutic modulation in PVR.

PVR is characterized by the migration and proliferation of RPE cells, leading to membrane formation, retinal detachment, and subsequent vision loss 1. Although trans pars plana vitrectomy remains the mainstay of treatment 5, the surgery itself is a risk factor for PVR, and no pharmacologic therapies are currently available 4, 6. Flavonoids like tricin have shown promise in inhibiting tumor cell spread, including oral cancer, ovarian cancer, and colon adenocarcinoma, often through apoptotic mechanism 17, 18, 36. These properties suggest that tricin may have therapeutic value in ocular proliferative disorders such as PVR.

In conclusion, tricin significantly suppresses the migration and invasion of ARPE-19 cells. This effect is mediated, at least in part, by the downregulation of CYP1A1 and RUNX2 expression through inhibition of the STAT3 signaling pathway (Figure 5D). These findings highlight tricin as a potential therapeutic candidate for RPE-related proliferative diseases such as PVR. Further *in vivo* and clinical studies are warranted to validate the efficacy and safety of tricin in modulating RPE behavior and preventing PVR progression.

## Figures and Tables

**Table 1:** List of the top 10 most downregulated and top 10 most upregulated genes in RNA sequencing, ranked by the absolute value of fold change.

Gene	log2 fold change	p value	change
CYP1A1	-3.460162505	0.003018156	Downregulation
CYP1B1	-1.921902762	0.00112057	Downregulation
GPC6	-1.419605948	0.010848769	Downregulation
NTNG1	-1.418452253	0.005305735	Downregulation
KRT13	-1.270749297	0.041971781	Downregulation
STARD4-AS1	-1.167385649	8.89498E-05	Downregulation
NREP	-1.085688671	8.58754E-17	Downregulation
TENM4	-0.991374878	0.001167913	Downregulation
RBMS3	-0.964522921	0.009127265	Downregulation
CA9	-0.929789135	2.76455E-07	Downregulation
CHAC1	2.264259873	0.042522634	Upregulation
CXCL8	2.135206128	0.009997994	Upregulation
TRIB2	1.861222808	0.045464909	Upregulation
EGR1	1.658501408	0.004784584	Upregulation
PDGFB	1.472564263	0.001171762	Upregulation
EDN1	1.350499157	0.039759726	Upregulation
FSCN1	1.291184011	0.008711675	Upregulation
RP11-800A3	1.260838653	0.049684098	Upregulation
JUNB	1.210008303	0.000120374	Upregulation
UPK3B	1.145507049	0.005413204	Upregulation
